# Chronic Suppuration of the Antrum

**Published:** 1902-04-26

**Authors:** 


### CHRONIC SUPPURATION OF THE ANTRUM.

Discussing the subject of chronic suppuration of the antrum Dr. Herbert Tilley [1] says, in regard to prognosis, that if antral suppuration be uncomplicated by similar disease in the ethmoidal sinuses, by nasal polypi, or by such gross lesions as carious areas of the bony walls of the sinus, the prognosis as regards relief of symptoms is good, but one must speak with reservation when a cure or total cessation of discharge is being considered. He adds a statement which seems to us of considerable importance in regard to the influence which the general health of the patients may in some cases have upon the disease. Two of his patients were most positive that the discharge absolutely ceased when they went to the seaside, but returned when they resumed their city life, while a third said that he could at once increase the discharge by indulgence in alcohol, excessive smoking, or any disregard of the ordinary rules of health. This seems to us to be a matter of some interest, for in view of the little certainty held out of obtaining a complete cure, even by operation, many people might be tempted to submit to the sacrifice involved in making an entire alteration of their usual mode of life (and indeed might prefer to do so rather than submitting to operative measures), if by so doing they could hope to get rid of so distressing a condition.

The local treatment of the disease may be by drainage and irrigation or by the more radical operation of curetting. Drainage and irrigation is attained by making an opening, generally by means of a simple perforator or awl, through the socket previously occupied by a molar or second biscuspid tooth, and inserting a silver tube. Twice daily the tube should be removed and some simple antiseptic lotion, such as boracic or Condy's fluid, injected by means of a modification of Higginson's enema syringe. As the discharge diminishes in amount the irrigations are less frequently carried out, and when after an interval of a week no discharge returns with the lotion the tube may be removed and the case considered as cured. This may seem, as indeed it is, a slight operation, but it is not altogether free from risk. In three of Dr. Tilley's cases the alveolar puncture was followed by free arterial hæmorrhage from the mouth and nose. In several cases also the insertion of the tube was followed by intense neuralgia, but this was in each case relieved by the removal of the tube and the substitution of one of a smaller calibre, so that it would seem wiser always to use a tube appreciably smaller than the opening made.

The more radical operation involves the exposure of the anterior surface of the antrum through an incision made along the gingivo-labial fold, extending from the level of the first molar to the canine tooth. A portion of the anterior wall of the antrum the size of a sixpence is cut out by means of a gouge and mallet, and the whole of the diseased lining of the sinus thoroughly curetted away. A counter opening of the same size is then made into the inferior meatus, and after disinfection the sinus is plugged, the end of the plug being passed through into the nostril and cut off level. The bucco-antral wound is then closed by fine sutures or allowed to close by granulation. The packing is removed in 48 hours, and the sinus is then regularly irrigated through the naso-antral opening for some three or four weeks, by which time the purulent discharge ought to have entirely ceased. Dr. Tilley says that the alveolar method should always be advised in the first instance, and he warns us that we must not confuse the terms radical operation and radical cure, for we cannot guarantee that the term "cure" will be justified by the results.

[1] Brit. Med. Jour., April 19.

